# Exploring prebiotic properties and its probiotic potential of new formulations of soy milk-derived beverages

**DOI:** 10.3389/fmicb.2024.1404907

**Published:** 2024-07-10

**Authors:** Ananya Rana, Neetu Kumra Taneja, António Raposo, Sehad N. Alarifi, Edite Teixeira-Lemos, Maria João Lima, João Carlos Gonçalves, Tejpal Dhewa

**Affiliations:** ^1^Department of Interdisciplinary Sciences, National Institute of Food Technology Entrepreneurship and Management, Sonipat, Haryana, India; ^2^CBIOS (Research Center for Biosciences and Health Technologies), Universidade Lusófona de Humanidades e Tecnologias, Lisboa, Portugal; ^3^Department of Food and Nutrition Science, Al-Quwayiyah College of Sciences and Humanities, Shaqra University, Shaqra, Saudi Arabia; ^4^CERNAS Research Centre, Polytechnic University of Viseu, Viseu, Portugal; ^5^Department of Nutrition Biology, Central University of Haryana, Mahendragarh, Haryana, India

**Keywords:** *Escherichia coli*, fermentation, *Lactiplantibacillus plantarum*, prebiotic activity score, soy milk beverages

## Abstract

**Introduction:**

The food and beverage industry has shown a growing interest in plant-based beverages as alternatives to traditional milk consumption. Soy milk is derived from soy beans and contains proteins, isoflavones, soy bean oligosaccharides, and saponins, among other ingredients. Because of its high nutritive value and versatility, soy milk has gained a lot of attention as a functional food.

**Methods:**

The present work aims to explore the prebiotic properties and gastrointestinal tolerance potential of new formulations of soy milk-derived drinks to be fermented with riboflavin-producing probiotic *Lactiplantibacillus plantarum* MTCC (Microbial Type Culture Collection and Gene Bank) 25432, *Lactiplantibacillus plantarum* MTCC 25433, and *Lactobacillus acidophilus* NCIM (National Collection of Industrial Microorganisms) 2902 strains.

**Results and discussion:**

The soy milk co-fermented beverage showed highest PAS (1.24 ± 0.02) followed by soy milk beverages fermented with *L. plantarum* MTCC 25433 (0.753 ± 0.0) when compared to the commercial prebiotic raffinose (1.29 ± 0.01). The findings of this study suggested that the soy milk beverages exhibited potent prebiotic activity, having the ability to support the growth of probiotics, and the potential to raise the content of several bioactive substances. The higher prebiotics activity score showed that the higher the growth rate of probiotics microorganism, the lower the growth of pathogen. For acidic tolerance, all fermented soy milk managed to meet the minimal requirement of 10^6^ viable probiotic cells per milliliter at pH 2 (8.13, 8.26, 8.30, and 8.45 logs CFU/mL, respectively) and pH 3.5 (8.11, 8.07, 8.39, and 9.01 log CFU/mL, respectively). The survival rate of soy milk LAB isolates on bile for 3 h ranged from 84.64 to 89.60%. The study concluded that lactobacilli could thrive in gastrointestinal tract. The sensory evaluation scores for body and texture, color, flavor, and overall acceptability showed a significant difference (*p* < 0.05) between the fermented probiotic soy milk and control samples. Soy milk fermented with a combination of *L. plantarum* MTCC 25432 & MTCC 25433 demonstrated the highest acceptability with the least amount of beany flavor. The findings of the study suggest soy milk’s potential in plant-based beverage market.

## Introduction

In the last 10 years, there has been a significant focus on research in all areas of food product development with the aim of developing newer, healthier food options to meet the evolving needs and demands of consumers. These demands have increased with urbanization; the current trend is focused research on functional and specialty beverages for newer products. Drinks are no longer just thought of as thirst quenchers in the modern world; consumers now look for specific functions in these drinks that fit into their lifestyle. These drinks may be functional in meeting various needs and lifestyles; they may increase vitality, combat aging, fatigue, and stress, or target particular illnesses. The market for these drinks is still growing (Sethi et al., 2016). The dairy industry has a long history of using particular strains of lactic acid bacteria to produce fermented products. Dairy yoghurt consumption has been linked to a number of health advantages, such as strengthened immune function, better nutrient absorption, and improved gut health. Lactic acid bacteria like *Lactobacillus delbrueckii* subsp. *bulgaricus* and *Streptococcus thermophilus*, which are frequently utilized as starter cultures in yoghurt production, are largely responsible for these benefits (Settachaimongkon et al., 2014; Narvhus and Abrahamsen, 2022). Non-dairy milk is experiencing a surge in popularity, driven by the expansion of the plant-based beverage market and the increasing demand for products catering to food intolerances (Tangyu et al., 2019). To address challenges with cow’s milk allergy, lactose intolerance, calorie concern, and the prevalence of hypercholesterolemia, one such important functional requirement is milk substitutes (Valencia-Flores et al., 2013). The lowest rates are found in adult white people from northern Europe, North America, and Australia; they range from 5% in the British population to 17% in Finland and northern France. Over 50% of people in South America, Africa, and Asia lack the ability to digest lactase, and in some Asian nations, this percentage approaches 100% (Lomer et al., 2008). Plant-based milk has become more popular because it contains no cholesterol or lactose. This makes it suitable for people with heart disease and lactose intolerance, as well as for everyone else (Ziarno et al., 2023). Considering these cultures’ well-established function in dairy fermentation, using them to make soy beverages that resemble yoghurt may result in a product experience that is comparable. This information can be used to improve the yield and quality of plant-based yoghurts, including soy-based varieties. One of the most extensively grown crops in the world is soy (Shurtleff and Aoyagi, 2013). In addition to being consumed by humans in a variety of forms such as soy flour, tofu, meat and coffee substitutes, and soy beverages, it is used as animal feed (Berk, 1992; Huang, 2008). Thus, investigating substitute plant-based foods for regular meals is beneficial (McClements and Grossmann, 2021; Alae-Carew et al., 2022). The primary goal of producers of plant-based dairy alternatives is to enhance the flavor and consistency of their goods in order to mimic and replace fermented milks with plant-based alternatives. Non-dairy aftertastes, issues with the bioavailability of plant proteins, and the content and bioavailability of minerals and vitamins are some of the obstacles that must be overcome (Mäkinen et al., 2015; Miriam et al., 2021; Leonard et al., 2022; Pua et al., 2022; Sugahara et al., 2022). These factors ought to be taken into account in subsequent studies investigating the application of dairy starter cultures to produce fermented soy beverages akin to yoghurt. According to research so far, these issues have been successfully resolved by using dairy starter cultures that have been carefully chosen (Blagden and Gilliland, 2005). Due to their potential health advantages and suitability for people with lactose intolerance or dairy allergies, yogurt-style fermented soy beverages have become increasingly popular among these substitutes (Madsen et al., 2021; Montemurro et al., 2021; Vieira et al., 2021). Dairy starter cultures have shown to be a viable method for producing these soy-based drinks in order to attain the required sensory qualities and guarantee consistent product quality.

Soy milk is made from soy beans and contains proteins, isoflavones, soy bean oligosaccharides, and saponins, among other ingredients. Researchers Onozawa et al. (1998) and Zava and Duwe (1997) with colleges Duwe discovered that eating isoflavones from soy beans lowered the risk of developing conditions like prostate and breast cancer. In addition to its cholesterol-lowering (Lee et al., 2005), anti-inflammatory (Lee et al., 2010), and antitumor activities (Zhang and Popovich, 2010) previous studies also reported that soyasaponin has a number of other advantageous properties, including lowering blood glucose levels (Benno et al., 1987). Both soy bean oligosaccharides (Wada et al., 1991) and raffinose (Benno et al., 1987), a sugar that is a component of soy bean oligosaccharides, have demonstrated effectiveness as prebiotics.

Prebiotics, as defined by the International Scientific Association for Probiotics and Prebiotics (ISAPP), are substances that selectively promote the growth and activity of beneficial microorganisms in the host, thereby conferring health benefits (Gibson et al., 2017). According to Chaturvedi and Chakraborty (2020), a diet rich in prebiotics plays a crucial role in modulating the gut microbiome through various pathways and helps in reducing a wide range of diseases, including cancer, inflammatory bowel disease (IBD), cardiovascular diseases (CVDs), and diabetes (Tanaka et al., 2006). Prebiotic consumption has also been demonstrated to increase the quantity of good bacteria in the gastrointestinal tract in a specific way. Hence, there is an overall improvement in health status associated with the consumption of these prebiotic beverages (Gyawali et al., 2019). While the majority of alternative beverages lack the complete nutritional profile of cow’s milk, they do contain active functional ingredients that contribute to overall well-being (Sethi et al., 2016). The prebiotic content in these beverages typically ranges from 0.3 to 6% (Khangwal and Shukla, 2019; Hao et al., 2021). Natural sources of prebiotics are abundant in various foods, including fruits, vegetables, and cereals like banana, tomato, asparagus, sugar beetroot, garlic, wheat, mushrooms, onion, artichoke, rye, milk, barley, chicory, honey, etc (Gebreus et al., 2008). Recent studies have highlighted the potential of several legumes to serve as valuable sources of prebiotics (Valero-Cases et al., 2020; Cichońska and Ziarno, 2021). Among these legumes, soy stands out due to its high protein content and fermentable carbohydrates. Soy beans contain prebiotic substances such as raffinose, stachyose, and verbascose (RFOs) (Wongputtisin et al., 2015). Because of its high nutritive value and versatility, soy milk has gained a lot of attention as a functional food. It has been demonstrated that consuming a variety of soy bean foods (Terada et al., 1999) and a soy bean milk-fermented product improves the intestinal environment. To get the desired sensory qualities, choosing the right starter cultures is essential. It’s crucial to take into account additional elements, like the availability of cultures created especially for plant-based beverages. Dairy starter cultures are widely used in industrial fermentation processes, such as the production of fermented milk, and are readily available on the market. Because these starter cultures are more widely available and possibly less expensive than cultures made especially for plant-based beverages, using them can be more economical. Furthermore, the fermentation of soy bean by probiotics such as *Lactiplantibacillus plantarum* subspecies enhances the nutritional value of the beans. This process increases protein digestibility and total phenolic content, while also inhibiting pathogen growth through the production of various antimicrobial compounds (Rui et al., 2017). The presence of different alpha-glucosidases in *L. plantarum* and *L. fermentum* makes them particularly well-suited for fermenting plant-based beverages like soy (LeBlanc et al., 2008). A prebiotic activity score (PAS) serves as a valuable tool for evaluating the efficacy of prebiotics in promoting the growth of pathogens, probiotics, or both. A high PAS indicates the effectiveness of prebiotic components in promoting probiotic growth and simultaneously preventing the colonization of pathogenic microorganisms in the presence of these prebiotic components.

Similarly to the previous example, a lower or negative PAS indicates that the prebiotic components facilitate pathogenic growth and do not contribute to the promotion of probiotic growth. Studies have investigated the prebiotic properties of various foods including mushrooms and red kidney bean water extract (Zakaria et al., 2018; Jayamanohar et al., 2019). However, to the best of our knowledge, there is a lack of research exploring beverages made from fermented soy milk. Our study aims to investigate the prebiotic qualities (prebiotic activity score) and probiotic survival rates in gastrointestinal conditions (acid and bile tolerance) of fermented soy milk. We utilized probiotic *Lactiplantibacillus plantarum* strains that produce riboflavin for this assessment. The aim of this study was to evaluate prebiotic activity and test *in-vitro* gastrointestinal tolerance of riboflavin enriched fermented soy milk with either or combination of the two riboflavin producing probiotic strains of *Lactiplantibacillus plantarum*, i.e., MTCC 25432 and MTCC 25433, and to perform sensory analysis of its developed product. We will examine the scientific underpinnings of this strategy, emphasizing the critical elements that affect the fermentation process and affect the properties of the finished product. Understanding the benefits and drawbacks of using riboflavin producing probiotic starter cultures can help us better understand how to improve the texture, sensory profile, and nutritional value of riboflavin enriched fermented soy milk that resemble yoghurt.

## Materials and methods

### Standard soy milk preparation

The preparation of soy milk was conducted following the standard method described by Narayan et al. (2021), with some modifications. Initially, soy beans (250 g) were soaked in distilled water and kept at room temperature, for 12 h. Then, the water was drained from the soy beans, and hydrated beans were manually peeled off to remove their test. The peeled beans were then placed in a mixer grinder (Bajaj Electronics, New Delhi, India) and ground for 5 min with 500 mL of distilled water. The resulting slurry was filtered using two layers of muslin cloth, and the final volume was adjusted to 1,000 mL with distilled water. The obtained soy milk was sterilized by autoclaving for 15 min.

### Microbial strains and culture combinations

All microbial strains tested are from the Microbial Type Culture Collection (MTCC) and National Collection of Industrial Microorganisms (NCIM) and have been previously isolated from food samples and extensively studied. *Lactiplantibacillus plantarum* MTCC 25432, *Lactiplantibacillus plantarum* MTCC 25433, and *L. acidophilus* NCIM 2902 were cultured from 60% (v/v) glycerol stocks stored at −20°C and propagated in a Man Rugosa Sharpe (MRS) (HiMedia) broth, at 37°C, in microaerophilic conditions, for at least 48 h. The microbial load of the inoculum was determined spectrophotometrically and subsequently confirmed by microbial plating. Microbial cells were centrifugated and resuspended twice in sterile water, before being added to the drinks. For the prebiotic activity test, *Escherichia coli* ATCC 25911 was used, and it was propagated aerobically in Nutrient broth (Hi-Media) at 37°C, for 24 h. To calculate the prebiotic activity of *L. plantarum* strains MTCC 25432 and MTCC 25433 against pathogenic bacteria, *E. coli* ATCC 25911 was taken.

### Fermentations design

The plant-based drink samples were fermented independently by *L. plantarum* MTCC 25432 (C-2%), *L. plantarum* MTCC 25433 (B-2%), and *L. acidophilus* NCIM 2902 (F-2%), as well as by a microbial mix (BCF) containing an equal proportion of *Lactiplantibacillus plantarum* MTCC (Microbial Type Culture Collection and Gene Bank) 25432 (2%), *Lactiplantibacillus plantarum* MTCC 25433 (2%), and *Lactobacillus acidophilus* NCIM (National Collection of Industrial Microorganisms) 2902 (1%) strains of the aforementioned strains (Unpublished data-communicated as research paper). The cell load of inoculated bacteria was standardized at log 8 CFU/mL. Fermentation of the soy milk was carried out in 50 mL final volume and incubated for 24 h at 37°C. Non-inoculated soy milk served as control. Two biological replicates of each formulation were performed. Microbial growth and pH were monitored and analyzed at the beginning of the experiment, over the time period of fermentation for 24 h.

### CFU counting

For enumeration of *Lactobacillus* strains and *E. coli* counts, 1 mL of each fermented soy milk sample was aseptically transferred into a sterile tube containing physiological solution (9 g/L NaCl) for serial dilution. The diluted samples were then plated in duplicate.

*Lactiplantibacillus plantarum* MTCC 25432 and *L. plantarum* MTCC 25433 were counted on MRS (HiMedia, New Delhi, India) agar medium after incubation for a minimum of 24 h, at 37°C. *L. plantarum* NCIM 2902 was counted on MRS agar medium after incubation in the same conditions as lactobacilli. The microbial mix was enumerated on the agar medium and incubated in the same conditions. *E. coli* ATCC 29011 enumeration was performed on Nutrient agar medium (HiMedia, New Delhi, India) and incubated at 37°C, for 24 h.

### Measurement of pH changes

The pH was determined with a pH meter (Eutech Instruements, Thermo scientific pvt limited, New Delhi, India) at 20°C after appropriate calibration with three standard buffer solutions at pH 9.21, pH 4.00, and pH 2.00. pH measurements were performed in triplicate at three separate time points to monitor the fermentation process.

### Prebiotic activity

The prebiotic activity of plant-based drinks was assessed as previously described with slight modifications in terms of growing culture media and carbohydrate taken (Fissore et al., 2015). In this study, media used as controls were supplemented with 1% glucose (w/v). Fructo-oligosaccharides (FOS) from HiMedia (HiMedia, New Delhi, India) served as the positive control prebiotic. Bacterial type strains *L. plantarum* MTCC 25432 and MCC 25433, *L. acidophilus* NCIM 2902, and *E. coli* ATCC 29011 were utilized and propagated in MRS broth (HiMedia, New Delhi, India), in MRS with 0.05% glucose and in NA broth (HiMedia, New Delhi, India), respectively. Lactobacillus strains were grown at 37°C for a minimum of 48 h under microaerophilic conditions, while *E. coli* ATCC 29011 was grown aerobically for 24 h, at 37°C. The microbial inoculum concentrations were adjusted using a spectrophotometer to achieve a final concentration of log 6 CFU/mL. The prebiotic activity score (PAS) was calculated following the methods described in previous studies (Huebner et al., 2008; Marotti et al., 2012; Fissore et al., 2015).

**Figure 1:**
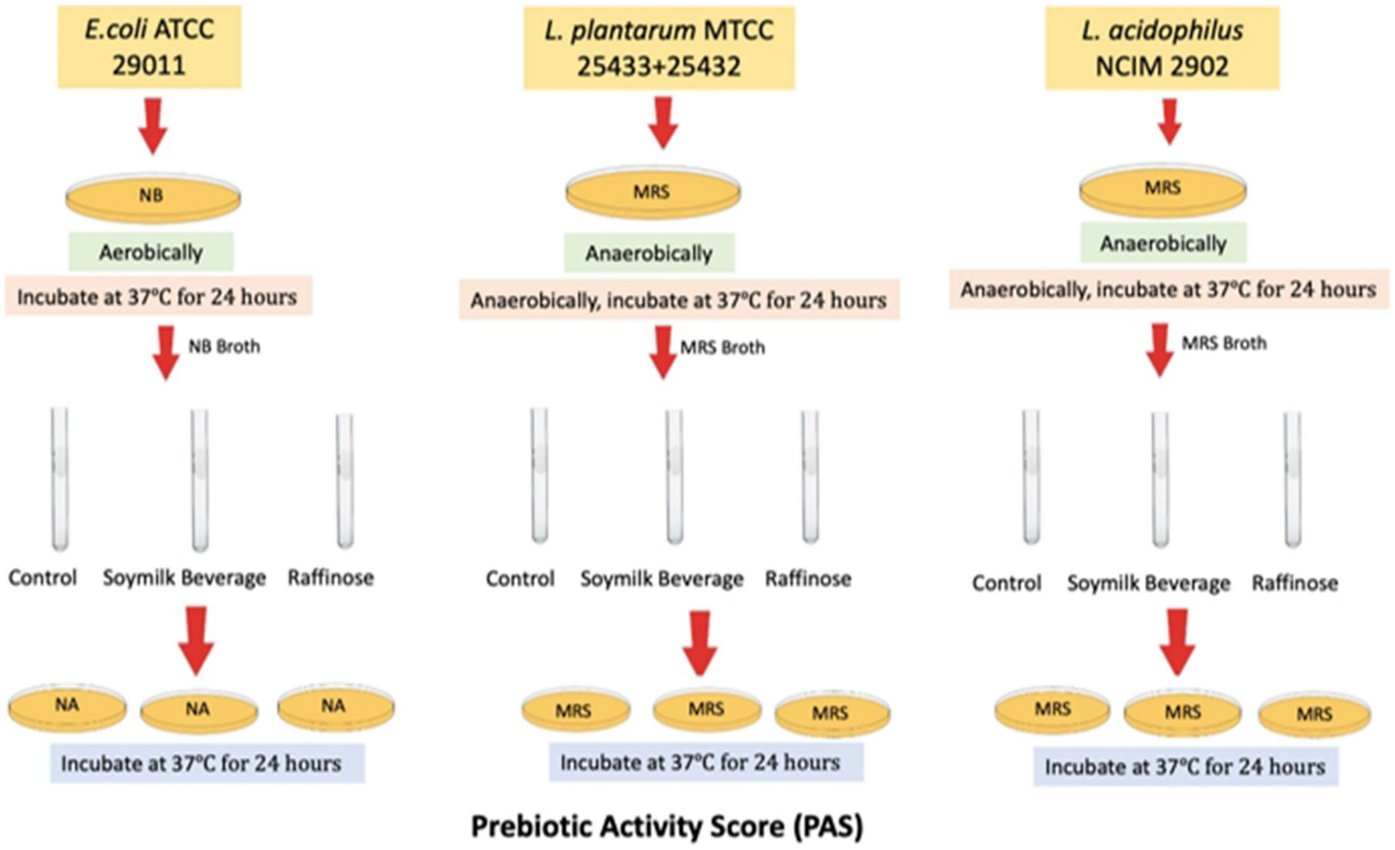
Flow-graph of method used for prebiotic activity score assay. Changes in cell density were calculated as differences in log 10 CFU/mL between the viable counts at 0 h and those at 24 h.

The PAS values for different for different Lactobacilli cultures were determined relative to *Escherichia coli* strains according to the method outlined by Huebner et al. (2007) as depicted in Figure 1. In order to quantify the prebiotic index and prebiotic activity score for novel *L. plantarum* strains grown on food matrix, the study aimed to measure the increase growth of *L. plantarum* strains MTCC 25432 & 25433, as probiotics, and *Escherichia coli*, as pathogenic enteric bacteria, to ferment soy milk beverages individually and co-fermented. Changes in cell density were calculated as the differences in log 10 CFU/mL between the viable count at 24 h and the viable count at 0 and 24 h.

PAS = (Changes in cell density on prebiotic – Changes in cell density of pathogen on prebiotic)/(Changes in cell density on glucose-Changes in cell density of pathogen on glucose).

### Determination of gastrointestinal tolerance/probiotic potential

The acid and bile tolerance assays were conducted to assess the probiotic potential of the stain, under gastrointestinal conditions.

For acid tolerance estimation the method outlined by Liong and Shah (2005) was employed. *Lactiplantibacillus plantarum* MTCC 25432 and MTCC 25433 probiotic strains were cultured in MRS broth and incubated at 37°C, for 16 h. Subsequently, 0.1 mL of the culture was adjusted to pH 3.5 and pH 2.0 using a 5 N HCl solution. The broth-containing cultures were incubated at 37°C, for 3 h. The viable count was measured every hour by spread plate technique using 10-fold serial dilutions in 0.1% saline water (Dallal et al., 2017).

Similarly, the bile tolerance was measured using the method described by Gilliland and Walker (1990). This method revived the strains in MRS broth for 24 h. Then, 1% of this active culture was added to 0.3% bile acid (Oxgall) containing MRS broth. MRS broth containing no bile acid was taken as control. The samples were incubated for 3 h, at 37°C. Samples were collected every hour for 3 h for viable count measurement of *Lactiplantibacillus plantarum* strains by spread plate technique for soy milk by serially diluting the samples in 0.1% saline solution.

Survival rates were calculated using the formula below (Thomrongsuwannakij et al., 2016):


$$\mathrm{Survival}\;\mathrm{rates}\;\left(\%\right)=\frac{\log N}{\log\mathrm{No}}\times 100$$

Where log N represents the logarithmic number of colonies present at the end of the test and log No is the logarithmic number of colonies present at the start of the test.

### Sensory evaluation

A panel of 30 semi-trained (ages 22–45 years) non-allergic panelists (staff and students) at NIFTEM, Sonepat, Haryana, evaluated the freshly made beverages based on their sensory perception. The sensory assessment took place in a room with 25°C temperature and incandescent white light. Each panelist performed the sensory task in groups of 10. The panelists were briefed on plant-based beverages, sensory parameters, scoring sheets, range scales, etc. prior to the evaluation. In total, four samples of soymilk beverages fermented with different probiotic Lactobacillus strain (s) were presented for sensory to evaluate the acceptability of sensory attributes of selection of most acceptable soymilk beverage. The sensory evaluation of fermented probiotic soymilk was conducted using a 9-point hedonic scale by a panel of semi-trained panelists consisting of 30 volunteers aged 22 to 45 years old. On a 9-point hedonic scale, the ratings were interpreted as follows: 9 = extremely like, 8 = very much like, 7 = moderately like, 6 = slightly like, 5 = neither like nor dislike, 4 = dislike slightly, 3 = dislike moderately, 2 = dislike very much, and 1 = extremely dislike. To evaluate the acceptability of sensory attributes such as mouthfeel, aroma, texture acceptability, and color and they were asked to give their response after tasting fermented and control samples and provide score to each and every sample as per their feel. The control sample and three fermented samples (soymilk) were coded using three-digit alpha-numeric coding. A 45–50 mL sample was served in transparent cups at temperature of 20°C. All panelist cleaned their oral cavity with drinking water before each serving. The main steps of this sensory evaluation were; (i) testing or evaluation of all samples; (ii) sum of sensory scores of each sample; and (iii) estimation of same values and scoring of the sample. The results were expressed as the average score across all the records obtained from the panelists’ evaluations.

### Statistical analysis

The experimental results were analyzed using Microsoft Office Excel 2010 for initial data processing. Subsequently, the differences between the groups were further analyzed by one-way analysis of variance (ANOVA). All experiments were performed in triplicate. One-way ANOVA (IBM SPSS Statistics Data Editor, Edition 19.0) was used for statistical analysis in order to examine the average values of the effects of various cultures on the growth of bacteria and the prebiotic activity score. At the 95% confidence level, *α* = 0.05 was used to evaluate all significant differences between means. To compare any significant differences between samples, ANOVA and Duncan’s multiple range test were utilized; *p* < 0.05 was considered statistically significant.

## Results and discussion

### pH and enumeration of bacterial growth

#### pH changes for selected cultures

Serial dilution was performed immediately after sampling by using saline water. One milliliter (1 mL) of fermentation media was taken for serial dilution at tenth-fold dilution (10–4 to 10–8) using sterile saline water. The viable counts of bacterial cultures were enumerated by the spread plate method using MRS agar (Hi-Media®, New Delhi, India) in triplicate analysis. The results presented in Table 1 were reported as colony-forming units per milliliter suspension as logarithm of microbial counts [log (CFU mL^−1^)]. The MRS agar and nutrient agar were prepared according to the instructions. The combination of *L. plantarum* strains demonstrated the most significant reduction in pH after 24 h of fermentation in all six of the matrices examined. The growth of *Lactiplantibacillus plantarum* strains is highly influenced by the substrate and pH of the medium. According to the obtained data, pH was significantly (*p* < 0.05) decreased in glucose for all strains during the fermentation process when compared to soy milk and standard prebiotic raffinose. As a simple sugar, glucose is directly usable by *Bifidobacterium*, *E. coli*, and *L. plantarum*. The presence of organic acids, primarily lactic acid released into the medium by lactic acid bacteria (LAB) is associated with a decrease in pH, a crucial indicator of fermentation progress (Liu et al., 2016).

**Table 1:** pH modifications during fermentation in soy milk by different culture combinations.

Soy milk with cultures	0 h	6 h	18 h	24 h
SM + B	6.06 ± 0.15 ^a^	4.56 ± 0.32 ^b^	4.18 ± 0.20 ^bc^	3.5 ± 0.50 ^c^
SM + C	6.00 ± 0.00 ^a^	4.72 ± 0.29^b^	4.40 ± 0.20 ^b^	3.00 ± 0.00 ^c^
SM + F	6.46 ± 0.41 ^a^	5.10 ± 0.00 ^c^	4.80 ± 0.20 ^d^	4.12 ± 0.02 ^e^
SM + BCF	6.03 ± 0.05 ^a^	4.53 ± 0.35 ^b^	4.00 ± 0.00 ^c^	3.11 ± 0.05 ^d^
SM + *E. coli*	6.43 ± 0.05 ^a^	5.86 ± 0.05 ^ab^	5.50 ± 0.50^bc^	5.01 ± 0.02 ^c^
Raffinose	6.03 ± 0.05 ^a^	5.03 ± 0.25 ^b^	4.48 ± 0.25 ^c^	4.50 ± 0.30 ^c^

SM + B = Soy milk fermented with *L. plantarum* MTCC 25432; SM + C = Soy milk fermented with *L. plantarum* MTCC 25433; SM+F = Soy milk fermented with *L. acidophilus* NCIM 2902; SM + BCF = Soy milk co-fermented with *L. plantarum* MTCC 25432 & 25433; SM + *E. coli* = Soy milk fermented with pathogenic *E. coli* ATCC 25911. Means with same letter are not significantly different. Different letters in same column and row indicate significant differences (*p* < 0.05).

The pH and nutrient of soy milk matrix supported its growth and the most favorable outcome was explicitly obtained in the soy milk fermented with combinations, with a pH decrease from 6.03 ± 0.05 to 4.53 ± 0.20 within 6 h. During the fermentation process with lactic acid bacteria, the beverage must rapidly become acidic to inhibit the growth of spoilage microbes. This capability was gone after 24 h; in fact, *L. plantarum* MTCC 25432 & MTCC 25433 did not achieve the highest acidification at the end of fermentation in any of the combinations investigated and the differences between them were insignificant (*p* < 0.05). With the exception of *E. coli*, all samples for *L. plantarum* and *L. acidophilus* strains showed a greater pH decrease after 6 h of fermentation. In meanwhile, after 12 h of fermentation, soy milk beverages fermented with pure cultures of lactic acid bacteria exhibit the largest pH reduction. Small pH variations occurred during the course of the 24-h *in vitro* fermentation process for both raffinose and unfermented soy milk, as expected. On the other hand, *E. coli* showed minimal pH decrease in all prebiotic samples (raffinose, non-fermented soy milk, fermented soy milk beverages, and glucose as control). While some *E. coli* strain can use glucose as their carbon source, others can use prebiotics as well (Hartemink et al., 1997). According to earlier research (Wolin, 1969; Roe et al., 1998), short chain fatty acids produced during fermentation contributed to the inhibition of *E. coli* at lower pH levels. Therefore, using soy milk may be better suited for co-fermentation using *L. plantarum* strains together with *L. acidophilus* NCIM 2902 starter culture. This outcome was consistent with other research that demonstrated certain *Lactiplantibacillus* strains favored the fermentation of oligosaccharides or disaccharides over monosaccharides (Ziarno et al., 2023). Hence, the result was consistent with the others since both strains’ pH values decreased, indicating that *L. plantarum* and *L. acidophilus* can use sources of carbohydrates instead of glucose. Soy milk upon fermentation makes available the soy bean oligosaccharides and raffinose, which is the constituent sugar of soy bean oligosaccharide, have exhibited efficacy as prebiotic. This further shows that prebiotic activity score of soy milk beverages is higher than that of glucose and raffinose.

### Growth of *Lactiplantibacillus plantarum* MTCC 25433 & 25432, and *Escherichia coli* ATCC 29011 on soy milk beverage as substrate

A prebiotic substrate should have the ability to specifically promote the growth and/or activity of gut bacteria linked to health and wellness. Consequently, strains of *Lactiplantibacillus* were studied for their increase in population cell number after growing for 24 h on glucose, raffinose, and soy milk beverages as substrate. The growth of *E. coli* 29011, which was selected to represent the enteric portion of the commensal flora, was further investigated using the same approach.

Table 2 presents the comparison of the growth of the pathogen *E. coli* and probiotic *L. plantarum* within 24 h in various prebiotic components, including beverages and standard sugar. In terms of microbial growth in soy milk beverages, the combination of *L. plantarum* strains yielded the most favorable outcomes in fermented beverages. *L. plantarum* MTCC 25433 demonstrated the highest performance among the culture combinations in soy milk-beverages after 24 h, while the microbial mix was the most effective in both *L. plantarum* MTCC 25432 & 25433. The growth increment of the microbial mix surpassed log 4 CFU/mL as compared to *E. coli* and reached its peak values on soy milk drinks after 24 h. When selecting a prebiotic and probiotic combination, it is important to consider an organism’s growth rate on a particular carbon source because this will affect its ability to compete with other microflora organisms in the colon for carbon sources (Hopkins et al., 1998; Ziarno et al., 2023). According to the findings, *L. plantarum* MTCC 25433 & 25432 in combination with *L. acidophilus* NCIM 2902 grew on soy milk as a carbon source results in the highest prebiotics score of 1.24 at 24 h (*p* < 0.05). Compared to *E. coli*, *L. plantarum* mix exhibits a high rate of cell proliferation during a brief incubation period of 24 h. To use prebiotics, lactic acid bacteria and other bacteria require the presence of a particular hydrolysis and transport system (Wada et al., 1992). Therefore, variations in the prebiotics score can result from strains having these gene-coding transport systems present or absent. Soy milk beverages showed more its potential as prebiotics on novel *L. plantarum* probiotic strains compare with starter culture *L. acidophilus* NCIM 2902 and *E. coli* ATCC 29011 since it has higher score. These results were in agreement with the previous studies (Rubel et al., 2014) which proved that, the higher prebiotics activity score showed that the higher the growth rate of probiotics microorganism, the lower the growth of pathogen. These indices the more selective used the prebiotics by probiotics microorganism in relation to glucose and limited used of prebiotics in relation to glucose by pathogen microorganisms.

**Table 2:** Comparison of microbial content increase within 24 h, in media composed of different components, in various matrices.

		Microbial population (log CFU/mL)			
Sample	*L. plantarum* MTCC 25433	*L. plantarum* MTCC 25432	*L. plantarum* MTCC 25433 + MTCC 25432	*L. acidophilus* NCIM 2902	*E. coli* ATCC 29011
Control	8.05 ± 0.00 ^b^	7.97 ± 0.05 ^b^	8.29 ± 0.20 ^b^	7.78 ± 0.25	4.00 ± 0.05
Raffinose	7.96 ± 0.19 ^b^	8.02 ± 0.12 ^b^	8.10 ± 0.20 ^b^	7.91 ± 0.02	4.32 ± 0.19
Soy milk	9.02 ± 0.20 ^a^	8.90 ± 0.10 ^a^	9.56 ± 0.20 ^a^	7.90 ± 0.26	4.59 ± 0.20

The probiotic as well as pathogenic bacterial growth on the different matrices (Control, Raffinose, and soymilk) are represented in the table. Control is glucose (monosaccharide), raffinose is trisaccharide synthesized from galactose, glucose, and fructose, and soymilk is polysaccharide. Means with same letter are not significantly different. Different alphabets in same column indicate significant differences (*p* < 0.05).

### Prebiotic activity score (PAS)

As summarized in Table 2, the comparison of the growth of the pathogen *E. coli* and probiotic *L. plantarum* within 24 h in various prebiotic components, such as beverages and standard sugar, was conducted. The growth of probiotics exceeded 7 log CFU/mL in all samples, while the growth of enteric pathogens was limited to 4.0 log CFU/mL. The higher the count of probiotics present in the human gut, the more beneficial impacts they will have in the human gut. Among all samples, soy milk fermented with *Lactiplantibacillus plantarum* MTCC 25433 & 25432 exhibited the highest probiotic growth (9.56 ± 0.04 log CFU/mL), followed by *Lactiplantibacillus plantarum* MTCC 25433 (9.02 ± 0.12 log CFU/mL). The prebiotics (oligosaccharides) from soy beans may be responsible for this enhanced growth. Moreover, the selective metabolic activity of the probiotic strains related to the concentration of sugars in soy beans, may contribute to the reduced growth of *Lactobacillus acidophilus* in soy milk beverages (Huebner et al., 2007). In each prebiotic component tested, the growth of the enteric pathogen *E. coli* ranged from 4.00 to 4.59 log CFU/ml. The limited growth of enteric pathogens may be attributed to the production of bacteriocins, which can compete with or kill disease-causing pathogens, as well as the competitive exclusion mechanism of probiotics (Fujisawa et al., 2017).

All the combinations of drinks exhibited an acceptable number of probiotics (>10^6^ CFU). According to FAO/WHO guidelines, for probiotics to positively impact the gut, a daily dose of 10^8^ CFU is necessary (Tripathi and Giri, 2014). In a study conducted by Taghizadeh et al. (2018), the total viable count of probiotic microbes in soy milk was 7.54 ± 0.06, 8.02 ± 0.01, and 7.00 ± 0.0 log CFU/mL, after 24 h. These results were obtained from *Lactobacillus acidophilus*, *Lacticaseibacillus paracasei*, and *Bifidobacterium lactis*. The authors concluded that the inclusion of the probiotic strains in soy milk improved the viability of the three probiotic bacteria. Another study by Battistini et al. (2018) found that *L. acidophilus* did not significantly grow in soy milk supplemented with prebiotics such as fructose oligosaccharide (FOS), inulin, and their combination. However, all samples had a viable cell count greater than 6 log CFU/mL. Therefore, these studies suggest that choosing the appropriate prebiotic source is crucial for promoting probiotic growth count. As long as the prebiotic maintains the desired count and other physicochemical properties, it can be added externally or as a component of the carrier matrix.

Table 3 presents the findings of the prebiotic activity score (PAS). A high PAS indicates the effectiveness of prebiotic components in promoting probiotic growth and suppressing pathogens in their presence. Conversely, a lower or negative prebiotic score suggests that probiotic growth is not supported and that colonizing pathogens is facilitated by the prebiotic components. The soy milk co-fermented beverage (1.24 ± 0.02) exhibited the highest PAS followed by fermented with *L. plantarum* MTCC 25433 (0.753 ± 0.0) when compared to the commercial prebiotic raffinose (1.29 ± 0.01). This indicates that the prebiotic components in soy beans present probiotic bacterial colonization and simultaneously inhibiting the survival of enteric pathogens, resulting in a favorable prebiotic score.

**Table 3:** Prebiotic activity score (PAS) of *Lactobacillus* strains (calculated using log 10 CFU/mL values) grown on different prebiotics.

Prebiotic source	*L. acidophilus* NCIM 2902	*L. plantarum* MTCC 25432	*L. plantarum* MTCC 25433	*L. plantarum* MTCC 25433+ MTCC 25432
Control	0.05 ± 0.00 ^c^	0.04 ± 0.02 ^c^	0.07 ± 0.00 ^c^	0.06 ± 0.00 ^c^
Raffinose	0.22 ± 0.00 ^b^	0.24 ± 0.04 ^b^	0.23 ± 0.00 ^b^	1.29 ± 0.00 ^b^
Soy milk	0.31 ± 0.00 ^a^	0.75 ± 0.05 ^a^	0.52 ± 0.08 ^a^	1.24 ± 0.01 ^a^

The prebiotic activity of monosaccharide (control), trisaccharide (Raffinose), and polysaccharide (soymilk) were evaluated to measure the prebiotic potential of different matrix. Each value represents a mean ± standard deviation. The alphabets in superscript across the column are significantly different at *p* < 0.05.

The variation in total PAS among different prebiotic sources may be attributed to the different metabolic capacities and activities of *L. plantarum* strains, resulting in higher prebiotic scores in soy milk beverages compared to raffinose. Previous research by Huebner et al. (2007) demonstrated that various probiotic strains require specific transport and hydrolysis systems to utilize different prebiotic components, thus affecting the PAS. These indicate the limited use of prebiotics in relation to glucose by pathogen microorganisms and the more selective use of prebiotics by probiotic microorganisms. When the prebiotic activity score is low or negative, it means that the growth of *Lactiplantibacillus plantarum* and *Lactobacillus acidophilus* on the prebiotic medium without carbon sources which are obtained from glucose is less favored during the same fermentation period. The fact that enteric strains grow less constrained by the availability of carbon sources on the medium than probiotic strains does not help explain the low prebiotics score (Rubel et al., 2014). The low PAS value yields obtained further demonstrates that *L. plantarum* as well as *L. acidophilus* demonstrated the ability to ferment all prebiotics selectively use carbohydrates (raffinose, glucose, and soy milk) during a 24-h incubation period. The ability of soy milk to ferment and support the growth of *L. plantarum* strains makes it effective as prebiotics after consumption. Despite having high growth rates, the individual bacterial strains received a lower score for fermentation time because *E. coli* growth was also high. Tsuda and Miyamoto (2010) report that during the initial stages of fermentation, *E. coli* has been observed to utilize lactose and monosaccharides instead of prebiotics. According to Gebreus et al. (2008), digestible dietary fiber is fully fermented by gastrointestinal tract microorganisms more easily than non-digestible fiber. Higher water-holding capacity and higher fermentability digestible fiber allowed bacteria to more easily break down the fiber (Marotti et al., 2012).

The prebiotics activity score indicates how much lactic acid bacteria can be supported by prebiotics.

The soy milk drinks scored much higher on the prebiotic activity scale. Thus, soy milk-based beverages could be a promising new source of prebiotics. Overall, each developed different probiotic culture-based soy milk beverages exhibited a positive and high PAS, suggesting that soy beans are an excellent matrix for carrying probiotics and promoting their growth while inhibiting the growth of harmful bacteria.

### Probiotic potential

#### Acid tolerance

The endurance of *L. plantarum* following 3 h of incubation in these conditions was used to confirm the probiotic bacteria’s tolerance to harsh gastrointestinal conditions, including acid and bile tolerance. Human digestion relies on the gut’s acidic pH, imposed by various gastric juices (Hayakawa et al., 1990). Additionally, the acidic environment inhibits the growth of different pathogenic microorganisms, thereby protecting the body from gastrointestinal illnesses (Lu et al., 2010). Therefore, a probiotic microbe should be able of withstanding and thriving in these acidic environments with elevated bile concentrations.

The probiotic survival rates from different fermented soy milk beverages at varying pH (2 and 3.5) and bile concentrations (0.3%) are displayed in Tables 4, 5, respectively. According to Sahadeva et al. (2011), a healthy probiotic should withstand a pH of 3.5 in normal gut conditions and a pH of 1.5–2 when fasting. However, the probiotic count typically declines when the pH drops from 3.5 to 2.0 or lower.

**Table 4:** Total plate count (log CFU/ml) for legume-based beverages for different culture combinations on MRS agars at different pH values of 2 and 3.5, for 3 h.

Total plate count (log CFU/mL)						
pH	Sample	0 h	1 h	2 h	3 h	Survival rate %
	Soy milk + F	8.13 ± 0.10 ^a^	7.89 ± 0.07 ^b^	7.45 ± 0.05 ^c^	6.23 ± 0.03 ^d^	76.62
	Soy milk+ B	8.26 ± 0.05 ^a^	7.75 ± 0.04 ^b^	7.14 ± 0.01 ^c^	6.11 ± 0.05 ^d^	73.97
2	Soy milk+ C	8.30 ± 0.10 ^a^	7.95 ± 0.00 ^b^	7.14 ± 0.01 ^c^	6.22 ± 0.00 ^d^	74.94
	Soy milk + BCF	8.45 ± 0.00 ^a^	7.79 ± 0.13 ^b^	7.23 ± 0.00 ^c^	6.99 ± 0.20 ^d^	82.72
	Soy milk + F	8.11 ± 0.08 ^a^	7.71 ± 0.01 ^b^	7.88 ± 0.12 ^c^	7.00 ± 0.00 ^d^	86.31
3.5	Soy milk+ B	8.07 ± 0.01 ^a^	7.83 ± 0.06 ^b^	7.65 ± 0.05 ^c^	6.98 ± 0.03 ^d^	87.25
	Soy milk+ C	8.30 ± 0.00 ^a^	8.02 ± 0.02 ^b^	7.89 ± 0.00 ^c^	7.12 ± 0.02 ^d^	85.78
	Soy milk + BCF	9.01 ± 0.01 ^a^	8.85 ± 0.04 ^b^	8.01 ± 0.02 ^c^	7.78 ± 0.00 ^d^	86.35

Alphabets in small letters (a–d) over the bar signify that the mean values at different hour for the same beverage are significantly (*p* < 0.05).

**Table 5:** Total plate count (log CFU/mL) for soymilk-based beverages for different culture combinations at 0.3% bile concentration, for 3 h.

		Total plate count (log CFU/ml)				
Bile conc.	Sample	0 h	1 h	2 h	3 h	Survival rate (%)
	Soy milk + F	8.06 ± 0.02 ^a^	7.91 ± 0.01 ^b^	7.56 ± 0.01 ^c^	7.11 ± 0.10 ^d^	88.87
	Soy milk+ B	8.06 ± 0.02 ^a^	7.87 ± 0.01 ^b^	7.63 ± 0.01 ^c^	6.99 ± 0.05^d^	86.72
	Soy milk+ C	8.14 ± 0.01^a^	7.91 ± 0.06^b^	7.76 ± 0.0.03 ^c^	6.89 ± 0.01 ^d^	84.64
0.30%	Soy milk + BCF	8.56 ± 0.01 ^a^	8.21 ± 0.00 ^b^	8.00 ± 0.00 ^b^	7.67 ± 0.02 ^c^	89.6

*Alphabets in small letters (a–c) over the bar signify that the mean values at different hour for the same beverage are significantly (*p* < 0 .05).

During the initial inoculation phase (the zeroth hour), all fermented beverages exhibited a good count ranging from 9.01 to 8.07 log CFU/mL, for both pH concentrations. However, after a 1- to 2-h incubation period, the growth of probiotics drastically decreased, indicating the detrimental effects of high gastric acidity and harsh pH on probiotics. It is likely that the high acidity and harsh pH conditions caused disruption of probiotic cell membranes, leading to cell disintegration due to bile salts and digestive enzymes (Fiocco et al., 2019).

Similar observations were reported by Reale et al. (2015), who cultured *L. casei* isolated from wine, at pH 2.5 for 2 h, and observed that cell multiplication resumed after 24 h at pH 3.5. However, growth was not observed at pH 1.5, indicating that lethal effects of low pH. Furthermore, Guan et al. (2017) demonstrated that isolated probiotic fermented milk exhibited a higher survival rate at pH 4 than at pH 2.5. Similarly, Rossi et al. (2018), found that LAB isolates from soy milk appeared to have varying tolerances to different pH levels. Despite the decrease in cell counts at pH 3.5, the decrease in cell was not sufficiently high to eliminate every cell. The survival rate for fermented soy milk under pH 3.5 was higher (ranging from 85.72 to 87.25%) compared to pH 2 (ranging from 74.94 to 82.72%).

Similar findings were documented by Kumari et al. (2022), who investigated the survivability of *Lacticaseibacillus rhamnosus* NCDC953 and *Limosilactobacillus reuteri* NCDC958, in soy milk. They demonstrated that both strains could withstand acidic environments, with pH 3 revealing higher cell viability than pH 2. The presence of sugars that produce ATP to facilitate proton exclusion may contribute to the enhanced viability of some probiotics in acidic environments (Kumari et al., 2022). Furthermore, the pH may not be low enough to cause breakdown of the cell membrane and disintegration by digestive juices.

According to the results in Table 4, there was a significant variation in probiotic survival between the two beverages at 0 and 3 h, with the soy milk co-fermented with both strains exhibiting the highest viability. However, after 3 h of exposure, all fermented soy milk containing various *Lactobacillus* strains managed to meet the minimal requirement of 106 viable probiotic cells per milliliter at pH 2 (8.13, 8.26, 8.30, and 8.45 logs CFU/mL, respectively) and pH 3.5 (8.11, 8.07, 8.39, and 9.01 log CFU/mL, respectively).

### Bile tolerance

Probiotics must possess the ability to tolerate bile salts to thrive in the small intestine, where they encounter these substances in the gastrointestinal tract (Wang et al., 2020). The trend observed in the acid tolerance test was consistent with the survival rate of bacteria in the presence of bile salt (0.3%). Following fermentation with *L. plantarum* MTCC 25433 (89.6%) and *L. plantarum* MTCC 25432 (86.72%), soy milk co-fermented with *L. plantarum* exhibited the highest survival rate (89.60%), after 3 h. The maximum growth (ranging from 8.06 to 8.56 log CFU/mL), as indicated in Table 5, was noticed at 0 h, and it subsequently significantly decreased for all of the beverages. In co-fermented samples containing *L. plantarum* MTCC 25433 & MTCC 25432 and *L. plantarum* MTCC 25433, the minimum viable counts were 7.67 ± 0.01 and 6.89 ± 0.50 log CFU/mL, respectively. However, according to FAO/WHO guidelines, these counts fell within the permissible range of 106–107 CFU/mL (Tripathi and Giri, 2014). Disturbances in cellular homeostasis and the dissociation of the lipid bilayer and integral protein of the probiotic cells’ cell membranes in the presence of bile acids may account for this decline in the viable rate. Rossi et al. (2018) found comparable outcomes in their studies. They showed that after incubation for 5 h, the viability decreased to 130–159% in a few isolates, and the survival rate of soy milk LAB isolates on 0.3% oxgall for 0 h ranged from 84.64 to 89.60%. The study concluded that lactobacilli could thrive in the high bile salt environment of the gastrointestinal tract.

However, for some *Lactiplantibacillus* strains, the decrease in viable counts may be due to modifications in the permeability of the cell membrane, which result in cell lysis, intracellular material leakage, and ultimately, cell death. Therefore, it can be concluded that the probiotic derived from the co-fermented soy milk beverage could withstand elevated gastrointestinal conditions, while maintaining a high viable total count.

### Sensory evaluation of the developed product

Thirty semi-trained panelists were asked to rate the developed beverages on sensory acceptability in order to determine which had the best mouthfeel and least amount of beany flavor. However, more panelists (more than 100) could shed even more light on the findings. Consumer preferences heavily rely on the sensory evolution of a product. Customers look for plant-based products that complement their values as well as ones that provide fulfilling culinary experiences. Millennials, who make up the majority of the customer demographic, are more likely than other age 25–34 to purchase dairy-and plant-based products. Based on the target audience, the panelist group was selected.

The response sheets from panelists were summed up to get the outcomes of sensory scores for quality attributes of fermented soymilk beverages. The overall evaluation of the fermented probiotic soymilk with *L. plantarum* MTCC 25432 and MTCC 25433 in combination was 8.06, indicating a very high level of acceptance. The order of samples were; Soymilk fermented with *L. plantarum* (MTCC 25433+ 25432) (very much like) > soymilk beverage fermented with *L. plantarum* MTCC 25433 (moderately like) > soymilk beverage fermented by *L. plantarum* MTCC 25432 (moderately like). The co-fermented beverage can therefore be concluded that out of all the beverages. The soymilk fermented with a combination of *L. plantarum* MTCC 25432 & MTCC 25433 demonstrated the highest acceptability with the least amount of beany flavor amongst all fermented beverages with different probiotic strain(s). This reduced and accepted flavor of the beverage could be attributed to the correct combinations of the two probiotic lactic acid bacteria helped in maintaining the nutritional and functional quantity at the same time. The Soymilk fermented with *L. plantarum* (MTCC 25433+ 25432) (8.06 ± 1.56) followed by soymilk fermented with *L. plantarum* MTCC 25433 (7.41 ± 1.45) showed reasonable compared to the control sample (6.86 ± 1.07), which indicates its suitability for human population that prefer plant-based beverages. The co-fermented soymilk beverage scored the highest value for texture acceptability (8.82 ± 1.16), flavor (8.76 ± 0.14), color (7.12 ± 0.44), mouthfeel (8.36 ± 0.56), and aroma (8.26 ± 1.04) amongst all the beverages, hence representing itself as a potential alternative for control (soymilk). The texture and color and other attributes for all the beverages were in a similar range (8.06–7.01). This might be because of the similar fermentation characteristics. The sensory evaluation scores for body and texture, color, flavor, and overall acceptability showed a significant difference (*p* < 0.05) between the fermented probiotic soy milk and control samples (Table 6).

**Table 6:** Sensory score of the soymilk beverages produced by different probiotics and overall acceptability of the developed beverages.

Attributes	Control/unfermented soymilk	Fermented probiotic soymilk (MTCC 25433 + 25432)	Fermented probiotic soymilk (MTCC 25433)	Fermented probiotic soymilk (MTCC 25432)
Appearance & texture acceptability	7.12^c^ ± 1.08	8.82 ^a^ ± 1.16	7.43 ^b^ ± 0.56	7.00 ^c^ ± 0.25
Flavor	5.32 ^d^ ± 0.51	8.76 ^a^ ± 0.14	7.63 ^b^ ± 0.60	6.72 ^c^ ± 0.51
Color	6.61 ^c^ ± 0.12	7.12 ^a^ ± 0.44	6.89 ^b^ ± 0.81	6.66 ^c^ ± 0.15
Mouthfeel	6.81 ^c^ ± 0.14	8.36 ^a^ ± 0.56	7.34 ^b^ ± 1.05	7.20 ^b^ ± 0.75
Aroma	7.21 ^d^ ± 1.07	8.26 ^a^ ± 1.04	7.98 ^b^ ± 0.05	7.50 ^c^ ± 40
Overall acceptability	6.86 ^c^ ± 1.07	8.06 ^b^ ± 1.56	7.41 ^b^ ± 1.45	7.01 ^a^ ± 1.02

*The alphabets in superscript across the rows are significantly different (*p* < 0.05). The most accepted according to the results provided by DMR-Test is fermented soymilk beverage with both *L. plantarum* MTCC 25433 + _25432 showing acceptability at very much like (8 on hedonic scale).

The sensory acceptance of soy milk beverages may have contributed to the fermentation with correct proportions inoculum gives palatable flavor while preserving its nutritional value (Mattick and Hand, 1969). For instance, Lopes et al. (2020) demonstrated that mixture-based beverages exhibited the best sensory characteristics in their investigation on the development of legume beverages from chickpea, lentil, and their mixtures. Therefore, it is possible to create beverages that are both nutrient-and flavor-rich by using the best possible concentrations of various legumes and inoculum concentrations and combinations (Ziarno et al., 2023). In a similar study, Winarsi et al. (2020) determined that, on a 5-point hedonic scale, the milk derived from sprouted red kidney beans with a germination time of 30 h had color, taste, flavor, and viscosity scores of 3.6, 3.21, 3.16, and 3.59, respectively.

The improvement in the overall acceptability of the fermented probiotic soy milk could be attributed to the production of EPS, high riboflavin content, and hydrolysis of the proteins, which may have positive effects on the consumers purchasing decisions. This suggests that probiotic and symbiotic drinks made from legumes can also be an acceptable beverage choice, with good consumer acceptability and an overall sensory score of more than six (out of 9 on hedonic scale).

The sensory evaluation was approved by NIFTEM Ethics Committee for Human Research (NECHR), NIFTEM-Kundli via Protocol no. 12/7L/NECHR/23 in July, 2023.

### Conclusion and future perspectives

Overall, the studies on beverages revealed a possible ability to specifically promote the growth of gut bacteria linked to health. The prebiotic potential legume-based beverages developed with soy beans were evaluated. Among the five formulations tested, the soy milk co-fermented with *L. plantarum* MTCC 25433 & 25432 exhibited a high and positive Prebiotic Activity Score (PAS), along with a desirable viable count (>10^6^ CFU/mL) of the probiotic bacteria *L. plantarum*. These indicate the limited use of prebiotics in relation to glucose by pathogen microorganisms and the more selective use of prebiotics by probiotic microorganisms. The fact that enteric strains grow less constrained by the availability of carbon sources on the medium than probiotic strains does not help explain the low prebiotics score. The low PAS value yields obtained further demonstrates that *L. plantarum* as well as *L. acidophilus* demonstrated the ability to ferment all prebiotics selectively use carbohydrates (raffinose, glucose, and soy milk) during a 24-h incubation period. The ability of soy milk to ferment and support the growth of *L. plantarum* strains makes it effective as prebiotics after consumption. For both strains, soy milk beverages that have been heat-pretreated and co-fermented have the highest prebiotic activity score because it has been found that:

- In relation to glucose, this beverage showed the highest specific growth rate of *Lactiplantibacillus plantarum* MTCC 25433 & MTCC 25432,

- When grown on this fraction and on glucose, *Lactiplantibacillus plantarum* MTCC 25433, *L. plantarum* MTCC 25432, and *L. acidophilus* NCIM 2902 were able to reach a similar population density,

- Regarding glucose, *Escherichia coli* ATCC 29011 showed a less pronounced rise in cell density on this beverage.

Furthermore, it received acceptable sensory scores, with a reduced beany mouthfeel. This meant it could be a new source of prebiotics for the non-dairy beverage industry. This indicates that it could serve as a promising new source of prebiotics for the non-dairy beverage industry.

The success of this formulation may be attributed to the dual functionality of plant-based drinks. These beverages can serve as carriers of probiotics while also supplying oligosaccharides (prebiotics) as energy sources for probiotics. Thus, soy milk beverages derived from legumes have demonstrated their potential to evolve into essential functional foods with desirable prebiotic properties, offering a range of health benefits.

Future research endeavors could focus on analyzing the volatile compounds associated with thoroughly examining the flavor profiles of these beverages. Additionally, exploring the utilization of symbiotic beverages as instant ready-to-reconstitute powders may be pursued by the non-dairy sector, aiming to enhance convenience and extend storage stability. While there is promise in the use of lactic acid bacteria as starter culture for soy beverage fermentation, there are a number of issues and limitations that must be taken into consideration. Given the growing demand for plant-based alternatives and consumer preferences, it is imperative to take into account emerging trends and develop new dairy starter cultures specifically made for fermenting plant-based raw materials. Understanding the science underlying this strategy and resolving the related obstacles will open the door to the development of delicious, nutritious, and high-quality plant-based products that fulfill consumers who are concerned about their health. This discovery could potentially open up the path for developing plant-based dairy substitutes that are not just more nourishing but also more delicious. Further the *in vivo* studies are required for testing the probiotic potential of their lactic acid bacteria to accurately evaluate its resistance to the acidic conditions of the stomach or bile tolerance, or by assessing their impact on complicated host functions such as immune development, metabolic functions, or the gut-brain interactions.

## Data availability statement

The original contributions presented in the study are included in the article/supplementary material, further inquiries can be directed to the corresponding authors.

## Ethics statement

The studies involving humans were approved by NIFTEM Ethical Committee on Human Research (NECHR), NIFTEM-Kundli via protocol number 12/7L/NECHR/23 in a meeting held on 19th July, 2023. The studies were conducted in accordance with the local legislation and institutional requirements. The participants provided their written informed consent to participate in this study.

## Author contributions

ARan: Conceptualization, Data curation, Formal analysis, Funding acquisition, Investigation, Methodology, Project administration, Resources, Software, Supervision, Validation, Visualization, Writing – original draft, Writing – review & editing. NT: Conceptualization, Data curation, Formal analysis, Funding acquisition, Investigation, Methodology, Project administration, Resources, Software, Supervision, Validation, Visualization, Writing – original draft, Writing – review & editing. ARap: Conceptualization, Data curation, Formal analysis, Funding acquisition, Investigation, Methodology, Project administration, Resources, Software, Supervision, Validation, Visualization, Writing – original draft, Writing – review & editing. SA: Conceptualization, Data curation, Formal analysis, Funding acquisition, Investigation, Methodology, Project administration, Resources, Software, Supervision, Validation, Visualization, Writing – original draft, Writing – review & editing. ET-L: Conceptualization, Data curation, Formal analysis, Funding acquisition, Investigation, Methodology, Project administration, Resources, Software, Supervision, Validation, Visualization, Writing – original draft, Writing – review & editing. ML: Conceptualization, Data curation, Formal analysis, Funding acquisition, Investigation, Methodology, Project administration, Resources, Software, Supervision, Validation, Visualization, Writing – original draft, Writing – review & editing. JG: Funding acquisition, Investigation, Methodology, Project administration, Resources, Software, Supervision, Validation, Visualization, Writing – original draft, Writing – review & editing, Conceptualization, Data curation, Formal analysis. TD: Conceptualization, Data curation, Formal analysis, Funding acquisition, Investigation, Methodology, Project administration, Resources, Software, Supervision, Validation, Visualization, Writing – original draft, Writing – review & editing.
